# Effect of Intra-Ply Hybrid Patches and Hydrothermal Aging on Local Bending Response of Repaired GFRP Composite Laminates

**DOI:** 10.3390/molecules25102325

**Published:** 2020-05-16

**Authors:** J. Jefferson Andrew, Vellayaraj Arumugam, Hom N. Dhakal

**Affiliations:** 1 Department of Aerospace Engineering, MIT Campus, Chromepet, Anna University, Chennai-600 044, India; jefferandrew@gmail.com (J.J.A.); arumugam.mitaero@gmail.com (V.A.); 2 Department of Mechanical Engineering, Khalifa University of Science and Technology, Masdar Campus, Masdar City 54224, Abu Dhabi, UAE; 3 Advanced Materials and Manufacturing (AMM) Research Group, School of Mechanical and Design Engineering, University of Portsmouth, Hampshire PO1 3DJ, UK

**Keywords:** patch repair, indentation test, acoustic emission (AE), polymer composites, hydrothermal aging

## Abstract

This study investigates the influence of intra-ply hybrid patches and hydrothermal aging on the indentation properties of patch repaired GFRP composites. Fabrics with various proportions of glass and Kevlar fibers were employed as the patches to achieve enhanced mechanical properties by hybridizing Kevlar and glass fibers together. Hydrothermal aging behavior of repaired composite laminates modified with water-resistant glass fibers in the outer layers was further investigated. Specimens were immersed in an environmental chamber containing seawater at temperatures of 30 (ambient), 50, and 70 °C until up to saturation. Damage mechanisms of repaired laminates were monitored using real-time acoustic emission (AE) technique. The experimental results showed that specimens repaired with 50G:50K patches offered superior performance than the virgin specimens. The hydrothermal aging effect on indentation behavior of the modified repaired specimens showed a considerable reduction in indentation properties, with higher strength retention exhibited by the repaired specimens modified with chopped glass fibers compared to the particulate fibers.

## 1. Introduction

The interest in employing patch repairs in laminated composites has considerably raised in recent years [1,2,3], especially in the automotive, aerospace and marine applications. Compared to mechanical fastener repair, adhesively bonded repair has improved structural integrity, excellent specific mechanical properties [4], superior fatigue behavior, low fabrication cost and high corrosion resistance [5,6]. Despite the above mentioned attractive attributes, there are a few inherent drawbacks that cause a substantial decrease in the residual strength and durability of such repaired laminates [7,8].

Carbon and glass fibers are commonly used as composite reinforcements in the transport sectors owing to their superior specific mechanical properties. On the other hand, these fibers have considerably low toughness and elongation properties. Repairing damaged areas (high-stress concentrated area) with these materials encourages premature failure of the laminate. Moreover, unidirectional patches are employed in most of the applications as they reveal high axial or in-plane mechanical response. However, unidirectional fibers have a low resistance to transverse or out-of-plane indentation loads [5,9,10,11]. This drawback can discard their use in some applications. Generally, for indentation loads, the bidirectional fibers are chosen over unidirectional ones due to their superior geometrical stability and ease of design [12]. These characteristics favor applying bidirectional fibers in patch repair applications.

Patch architecture and stacking sequence significantly influence the mechanical performance of patch repaired laminates [13,14]. The properties of patches can also be tailored through the hybridization technique to meet the exact requirements of the repair [15]. Usually, hybrid composites are manufactured by employing a brittle fiber and a ductile fiber in an appropriate proportion [16,17]. The addition of both brittle fiber (high stiff) and ductile fiber (high elongation) within the patches can increase the stiffness as well as the toughness of the repaired laminates [18]. In this regard, from the perspective of cost and availability, Kevlar fibers are considered to be an attractive choice [19,20]. Even though the significance of hybridization has been investigated by many authors, it is not yet clear how the mechanical performance of repaired laminates can be influenced by the hybrid patches. In this paper, the indentation response of adhesively bonded hybrid patch repaired composites is investigated to evaluate the importance of patch hybridization. The findings of this study can offer aerospace and other engineering applications with adequate references and relevant understanding.

Adhesively bonded repairs in service life can perhaps be exposed to various environmental service life (e.g., hydrothermal) conditions [19]. Exposure to hydrothermal aging can lead to the softening of the fiber-matrix interface and result in degradation of structures [16,17,21]. The water molecules concurrently influence the matrix system, fiber system, and their interface (plasticizer), as a result inducing areas of poor load transfer, which consequently decrease the mechanical properties [19,22,23]. The work carried out by Dhakal et al. [16] suggested that water absorption in laminates is controlled by three distinct events that comprise: (a) dispersion of water in the minute gaps flanked by polymer chains; (b) capillarity of the molecules into cracks at the interface linking matrix and fiber owing to reduced wetting; (c) dispersion of the water by cracks in the resin system taking place from the swelling of hydrophilic fiber system [8,24,25]. Daniel et al. [26] reported that the moisture uptake of composites increases with the rise in immersion temperature. They reported that the time required to attain the equilibrium plateau (saturation level) reduces by increasing the exposure temperature. This premature degradation of the composite is further reflected on the mechanical performance [27]. The weaknesses of repaired composite laminates due to moisture absorption can be rectified by treating the fiber used to perform a repair through physical or chemical treatments or by the application of coupling agents [28,29,30]. Alternatively, it can be done by the fabrication of repaired laminates with water-resistant fibers, which has a significant influence on the water diffusion response of the resulting laminates [30,31,32,33,34], thus facilitate an appropriate balance in cost and repair performance. To confirm the long-standing reliability and durability of patch repairs, the influence of environmental and mechanical loading should be considered in the design procedure. There is very little research work available examining the effect of aging condition on the mechanical behavior of repaired laminates [14].

Frequently, repaired laminates may be vulnerable to damage from non-transient transverse loads that can be characterized via quasi-static indentation tests [31,35]. The damage profile of a repaired laminate is considerably influenced by the loading and exposure conditions [14]. Repaired composite laminates exposed to environmental aging conditions exhibit several distinct failure modes under indentation [36]. Indentation load on patch repaired laminate can lead to considerable matrix cracking, rear side fiber breakage and debonding amid the weak parent-patch interfaces. The understanding of various damage mechanisms and their complex mutual interaction is essential to design an appropriate repair configuration and it is difficult to predict via numerical and analytical modeling [37]. Presently, the acoustic emission (AE) technique has been used by several researchers for the real-time monitoring of repaired laminates [38,39,40,41].

This present study is an extension of our previous work that investigated the mechanical response of damaged GFRP laminates repaired using hybrid patches [35]. This present study aims at investigating the effect of intra-ply hybrid patches and hydrothermal aging on the indentation properties of patch repairs in damaged GFRP laminates. The repaired specimens exhibiting the most favorable indentation response was found initially and employed for hydrothermal aging studies.

## 2. Experimental Procedure

### 2.1. Materials and Fabrication

Epoxy resin (LY556) and woven bidirectional glass fabric (600 g/m^2^), supplied by Mark Tech. Composites, Bengaluru, India, was employed to fabricate the parent laminates. The epoxy resin was mixed with hardener (HY 951) in a weight ratio of 10:1 to accelerating the curing process. The epoxy resin was reinforced with eight layers of glass fiber mats in a weight ratio of 1:1 by hand layup technique to fabricate into panels of dimension 500 mm × 500 mm with an average thickness of 4 ± 0.1 mm. A compression molding machine (load capacity 30 kN) was used to consolidate the hand-laid laminates at ambient temperature under a pressure of 5 MPa for about 12 h. A spacer plate of thickness 4 mm was positioned between the molds to retain a uniform thickness all over the panel. The nominal volume fraction of the fiber, matrix and porosity in the glass/epoxy laminates were 53.9, 39.5 and 6.6, respectively. A water jet machining process was employed to trim ASTM D6264-98 standard panels of dimension 150 mm × 100 mm from the fabricated laminates.

### 2.2. Repair Technique

Damage was induced by machining a circular hole of 20 mm diameter wholly through the thickness direction at the center of the GFRP specimens. Chopped fibers was reinforced into epoxy resin in a weight ratio of 1:1 and filled into the damaged region [42,43]. Throughout the repair process, the excess resin was wiped out using cotton soaked in an acetone. The damaged region of the GFRP specimens were repaired using square patches of sides 60 mm. The nominal thickness of the repaired region was 5.8 ± 0.1 mm. Patches with five various combinations of glass and Kevlar fibers were fabricated, a two homogeneous patches (glass fabric (100G) and Kevlar fabric (100K)) and three intra-ply hybrid patches (75G25K, 50G50K and 25G75K, where the amount of glass and Kevlar in the warp and fill direction are 75:25, 50:50 and 25:75, respectively). Detail fabrication procedure is explained elsewhere [39].

### 2.3. Preparation of Water Resistant Repair Specimens

**Figure 1:**
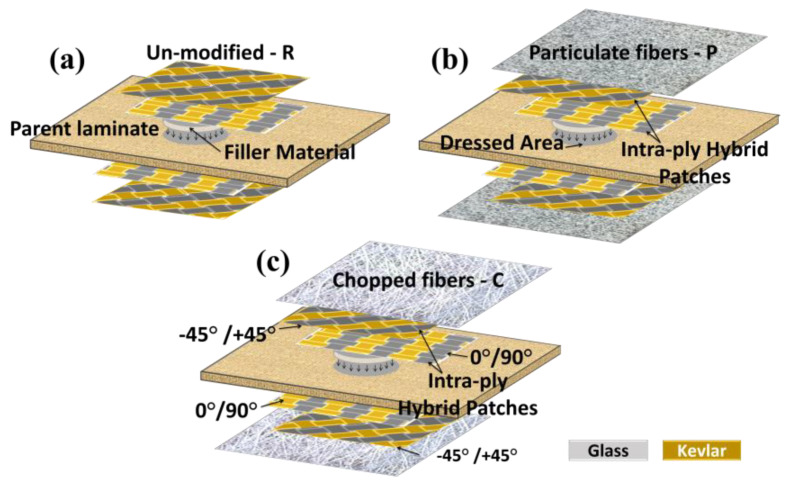
The structure of composites: (**a**) 50G50K repaired specimen; repaired specimens modified using (**b**) particulate fibers; (**c**) chopped fibers.

**Figure 2:**
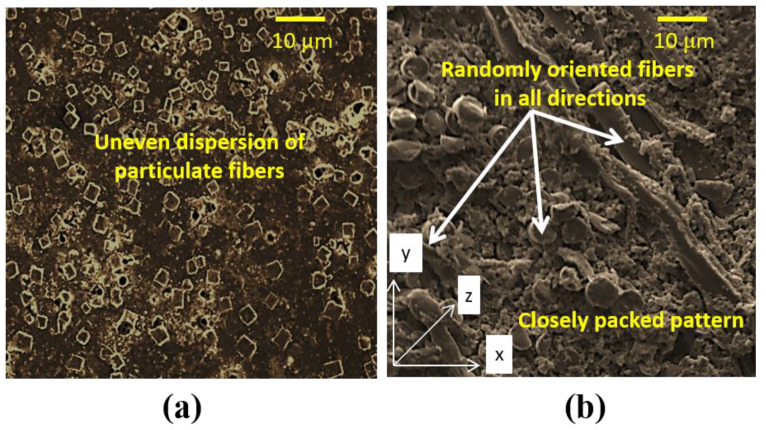
Surface morphology of particulate fibers and chopped fibers in (**a**) Type P and (**b**) Type C specimens, respectively.

The 50G50K specimens offered the superior indentation performance than virgin and other specimens; so, they were employed to perform hydrothermal aging studies. The particulate glass fiber of 175–250 µm size and chopped strand glass fiber with 4–6 mm length supplied by Mark Tech. Composites, Bengaluru, India was used for the fabrication of water-resistant repair specimens. They were added in the epoxy at a weight ratio of 1:1 and bonded over the repaired site as depicted in Figure 1. The main purpose of using water-resistant glass fibers in the form of particulate and chopped strands is to obtain layers of minimum thickness. Modifying the repaired specimens with these water-resistant materials increased the average thickness of the specimens to about 0.175 mm. Figure 2 shows the surface morphology of particulate fibers and chopped fibers in Type P and Type C specimens, respectively. All the specimens were post-cured at 50 °C for 2 h. Table 1 presents the configuration code of various specimens considered in this paper.

### 2.4. Environmental Aging Studies

To study the effect of hydrothermal aging, water diffusion behavior and their influence on indentation behavior of the repaired specimens were examined together with the consequence of elevated temperatures (50 and 70 °C), comparing the results with those acquired at ambient temperature (30 °C) [32,34]. The processed specimens were immersed in an environmental chamber containing seawater (RH relative humidity of 90%) at 30, 50 and 70 °C until up to saturation (1600 h). Here, the maximum temperature for the hydrothermal aging of different specimens was selected to be 70 °C, near to the boiling point of water 100 °C. Seawater was employed for immersion, to accelerate fiber–matrix interface debonding (e.g., effect of acid or NaCl content) [43]. The specimens were periodically taken out and weighed using a weighing machine (precision of ±1 mg) in 1 min, after drying the surface of the specimens with a tissue paper. The weighing process of the aged specimens was stopped once weight gain attained saturation stage, a stage when the moisture content or weight gain was almost constant. The weight gain was estimated using Equation (1) as follows:

M_t_ = (W_t_ − W_0_)/W_0_ (1)

where, M_t_, W_0_ and W_t_ are the moisture uptake, mass before and during aging, respectively. The Fickian diffusion coefficient was estimated by Equation (2):

D = π × (k × h/(4 × M_t_))^2^ (2)

Here, M_t_ is the moisture content at saturation, h is the thickness and k is the slope of the initial linear region of M_t_ = f(√t/h), as calculated by Equation (3):

M_t_ = (M_2_ − M_1_)/(√t_2_ − √t_1_) (3)

### 2.5. Quasi-Static Indentation Test with AE Monitoring

**Figure 3:**
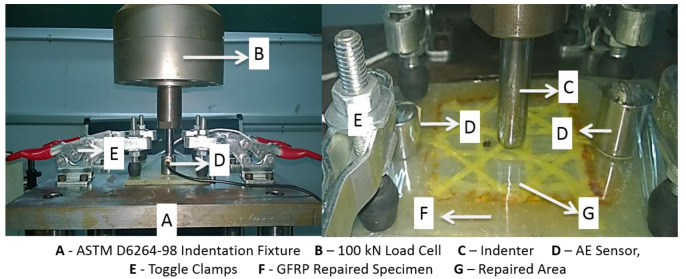
Glass/epoxy repaired specimen clamped in ASTM D 6264-98 indentation fixture.

A 100 kN Tinus Olsen UTM (Horsham, PA, USA) was employed to conduct the cyclic indentation tests at a cross head speed of 0.5 mm/min. Cyclic indentation load was applied sequentially at incremental displacement phases as per the ASTM D6264-98 standard. The specimens were clamped on the fixture as shown in Figure 3 and loaded using a hemispherical-faced indenter of diameter 12.7 mm. The damage, toughness and permanent displacement propagation observed from the mechanical tests are helpful to assess the balance of different indentation properties experienced in the specimens owing to the role of hybridization. The above-mentioned properties cannot be assessed from a monotonically increasing load and thus cyclic indentation loads were applied to perform this estimation from each load-displacement curve.

The damage profiles of the repaired laminates were monitored using a Physical Acoustics Corporation (PAC) Acoustic Emission device with a sampling frequency of 4 MHz. Wide-band AE sensors (100–900 kHz) were employed to capture the stress wave signals produced during the mechanical tests. Two AE transducers were mounted 50 mm symmetrically in the opposite direction to the damage spot using high sealant vacuum grease (silicone grease). The amplitude threshold was set to 40 dB. The pencil break test method, according to the ASTM E976-10 standard was employed to measure the wave velocity (3146.3 m/s).

## 3. Results and Discussions

### 3.1. Effect of Hybridization and Fiber Volume Fraction

**Figure 4:**
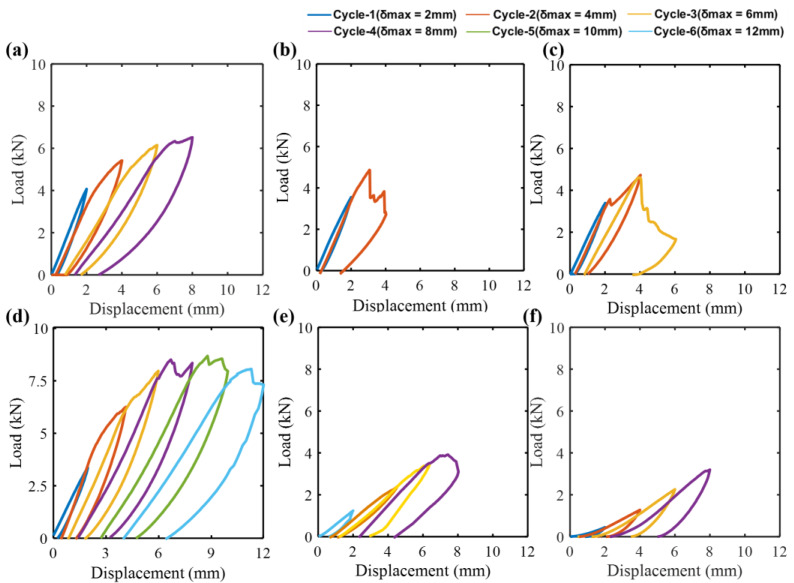
Load versus displacement plots of (**a**) Virgin, (**b**) 100G, (**c**) 75G25K, (**d**) 50G50K, (**e**) 25G75K and (**f**) 100 K specimens.

**Figure 5:**
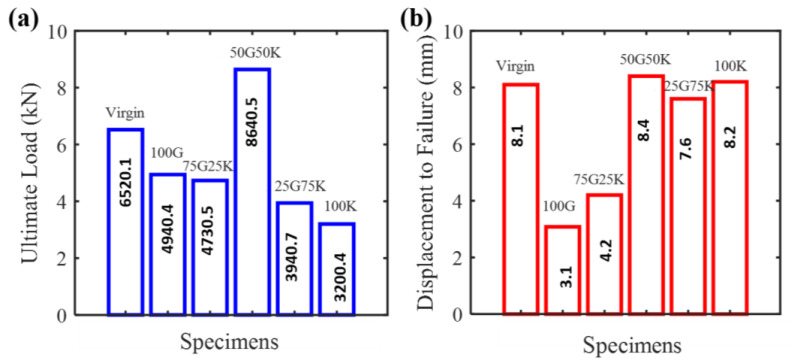
(**a**) Ultimate load and (**b**) ultimate displacement to failure of different specimens.

Load-displacement plots of different specimens subjected to cyclic indentation tests are shown in Figure 4. The loading was stopped when critical penetration (ultimate failure) was observed. Following the test results, the ultimate load and displacement of different specimens are shown in Figure 5. Within the scope of this study, for the comparison of the indentation properties, the virgin specimens were considered as the reference. The residual ultimate load is a promising parameter to rank the performance of various repaired specimens. It can be noticed that the ultimate load changed considerably with the proportion of glass and Kevlar fibers. Specifically, the 50G50K specimens exhibited a higher load carrying capability than the virgin specimens. The 100K specimens showed the highest loss at around 50% of the virgin ones. This critical decrease in the ultimate load of 100 K specimens might be due to the poor adhesion of Kevel fibers to the epoxy matrix and presence of a weak hydrogen bond in the out-of-plane direction [37]. These interpretations are described further via damage, stiffness and permanent displacement progression parameters of various specimens in the subsequent sections.

**Figure 6:**
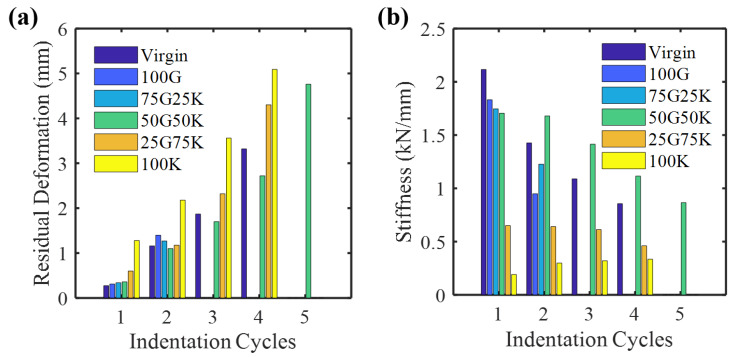
(**a**) Residual deformation and (**b**) stiffness progression of different specimens.

Figure 6 illustrates the residual deformation (permanent displacement) and stiffness for various specimens. The trend of residual deformation was the inverse of the stiffness at indentation cycle 1. The highest stiffness and the least residual deformation were exhibited by the virgin specimens. Among the repaired specimens, 100G exhibited the highest stiffness and the least residual deformation. This feature points out the brittle behavior of the glass fiber. In contrast, the least stiffness and the highest residual deformation was exhibited by 100K, demonstrating that it endured the load in a ductile manner owing to the higher elongation character of the Kevlar. The hybrid specimens (75G25K, 50G50K and 25G75K) showed a response amid 100G and 100K: this shows that increasing the proportion of Kevlar decreased the stiffness and caused them to respond in a ductile manner.

**Figure 7:**
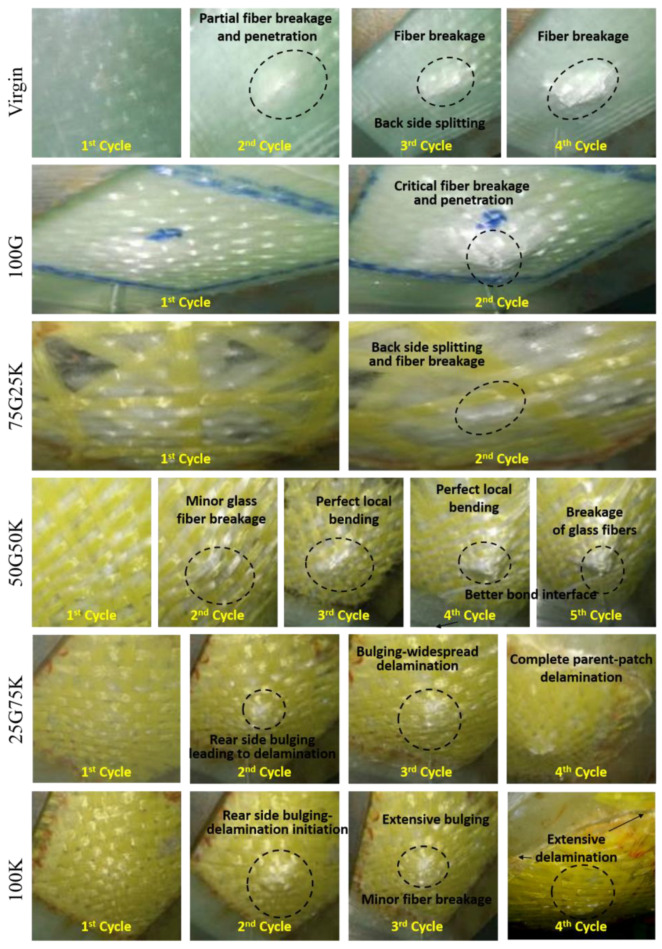
Photographic images of different specimens at various indentation cycles.

At higher indentation cycles, the virgin, homogeneous and hybrid repaired specimens exhibited a different response. This signifies that the failure mechanism liable for absorbing the mechanical energy was different at a higher indentation cycle. The 50G50K surpassed other repaired specimens by limiting the damage growth (Figure 7). Similar behavior can also be observed in residual deformation and stiffness progression plot in Figure 6. The indenter entirely penetrated the 100G and 75G25K at indentation cycle 2 (Figure 7). The repaired specimens with a higher volume fraction of glass fibers were more rigid. These specimens showed a brittle fracture via fiber breakage and rear face splitting. Furthermore, virgin specimens showed a brittle fracture, whereas 50G50K, being more ductile, induced ultimate failure at high displacement [42]. These interpretations clarify why 50G50K showed higher stiffness and lower residual deformation than virgin, 100G and 75G25K at cycle 2.

Throughout the indentation tests, 25G75K and 100K exhibited the lowest stiffness and the highest residual deformation. Compared to the cycles 2 and 3, the difference in residual deformation and stiffness between virgin and 50G50K were higher at cycle 4. Due to parent-patch interface delamination, 100K and 25G75K with higher Kevlar fives, exhibited ultimate fracture at a smaller displacement than 50G50K (Figure 7). On the other hand, the 50G50K yielded the best balance among rigidity, rear side fiber breakage and delamination at cycle 4. This recommends that for the ultimate fracture, the load has to be additionally increased, emphasizing the superior indentation performance. Thus, rather than the other specimens, the maximum displacement was considerably higher for the 50G50K. Since the 50G50K exhibited a more favorable mechanical performance, they were employed to perform hydrothermal aging studies.

### 3.2. Effect of Hydrothermal Aging

#### 3.2.1. Moisture Uptake Behavior

**Figure 8:**
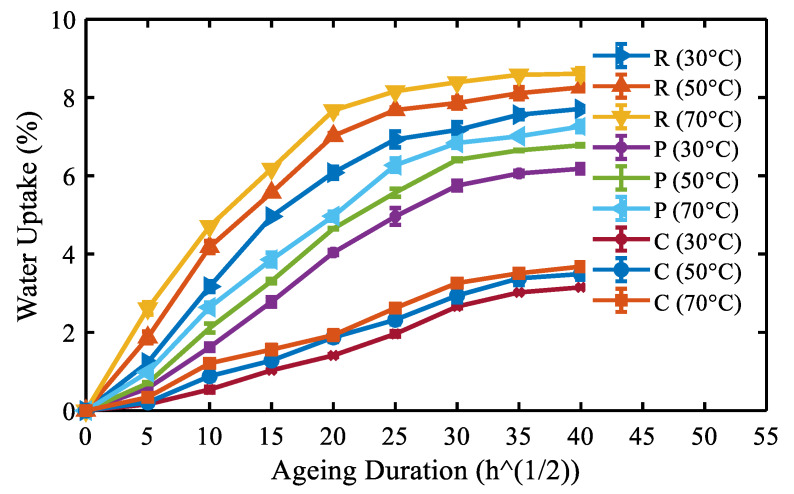
Water uptake curves of different composite specimens.

For engineering applications in the outdoor services of repaired laminates, it is vital to examine the influence of hydrothermal aging environment on the mechanical behavior of repaired composites. Moisture uptake curves of different repaired specimens at various immersion temperatures are depicted in Figure 8. The curves depict the percentage of moisture absorption behavior evaluated from the Fickian law of diffusion, as measured in [44]. The summary of the water uptake behavior of different specimens is shown in Table 2. Figure 8 shows that the specimens absorbed seawater abruptly in the initial phase of aging up to a saturation state was reached. At all the temperatures, moisture absorption of Type R specimen was the highest, whereas the Type C specimens exhibited the lowest. The specimens protected using particulate fibers (Type P) exhibited a moisture absorption behavior between Type R and Type C.

Based on the water-resistant behavior, various specimens can be ranked from low to the high order as: Type R < Type P < Type C. However, in all the specimens, the increase of immersion temperature reduced the duration required for attaining the saturation level in weight gain, which was characterized by an abrupt rise in the slope of the weight gain curves. The effect of immersion temperature on the saturation level was highly reflected in the case of Type R specimens. The value was constant about 7.17% at 30 °C after 756.25 days, 7.67% at 50 °C after 625 days and about 7.68% at 70 °C after 506.25 days. This variation in moisture absorption at 70 °C compared to 50 °C and 30 °C highlights that the Type R specimens would be highly degraded, with a substantial effect on the mechanical response. Furthermore, when the immersion temperature was raised from 30 to 70 °C, the diffusion coefficient and saturation moisture content increased significantly for Type R specimens.

**Figure 9:**
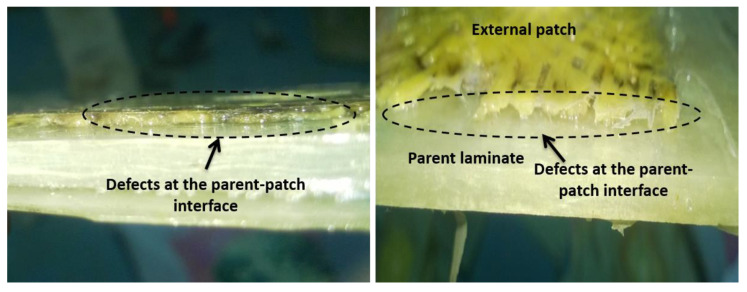
Photographic images showing defects at the parent-patch interfaces.

**Figure 10:**
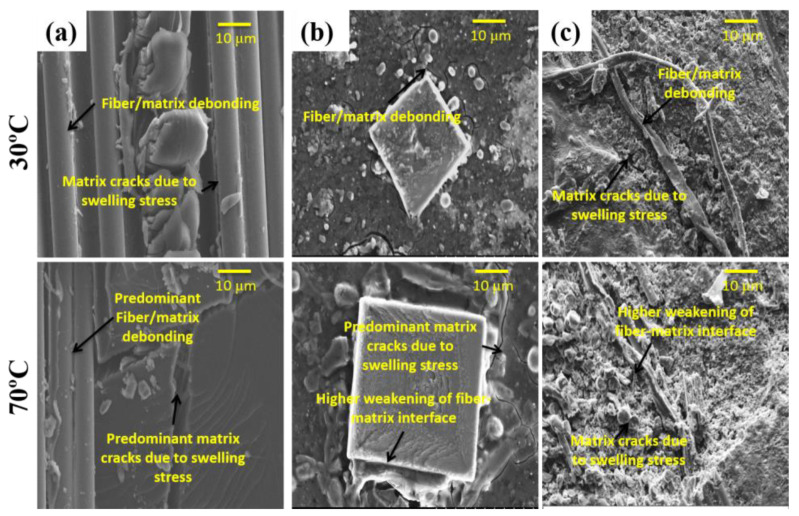
SEM images demonstrating particulars of the fractured surface of (**a**) Type R, (**b**) Type P and (**c**) Type C specimens.

In the case of Type R specimens, the higher water uptake was ascribed to the hydrophilic character of Kevlar and micro-flaws at the interface flanked by the parent and patch material (Figure 9), as the quantity of seawater absorbed by the glass fiber and matrix is very limited [16,45,46]. Specifically, the water uptake feature in Type R is controlled by fiber and the matrix system has an extremely hindering influence on the water absorption. This is a familiar event in Kevlar fiber-reinforced composite specimens, as Kevlar molecule is a well-known example of polar molecules that absorb higher moisture than the matrix system in which they are reinforced [14,30,47]. When the Type R specimens were exposed to aging, the hydrophilic Kevlar fibers bulged and induced micro resin cracks (Figure 10a). Following this, the capillarity phenomenon through resin cracks became active. This phenomenon resulted in premature fiber-matrix debonding [16,19,48].

The moisture uptake behavior of Type P and Type C specimens was observed to be much less than that of the Type R specimens. Among the Type P and Type C specimens, the later one showed the highest water-resistance property. This behavior in Type C was based on the fact that the external chopped fibers were inherently placed in a close-packed configuration in which the glass fibers acted as the protecting layer (Figure 1). Therefore, water penetration during hydrothermal aging was significantly decreased by modifying the repaired composites with chopped glass fibers. The particulate fibers were unevenly dispersed in the matrix compared to the chopped fibers. Owing to the agglomeration, addition of particulate fibers in the matrix decreases the fiber/matrix interfacial area. The particulate fibers agglomerates scattered in the polymer matrix, act as pores and result in stress concentration (induce cracks under loading), further increases the water penetration.

#### 3.2.2. Effect of Moisture Absorption on Quasi-Static Indentation Behavior

**Figure 11:**
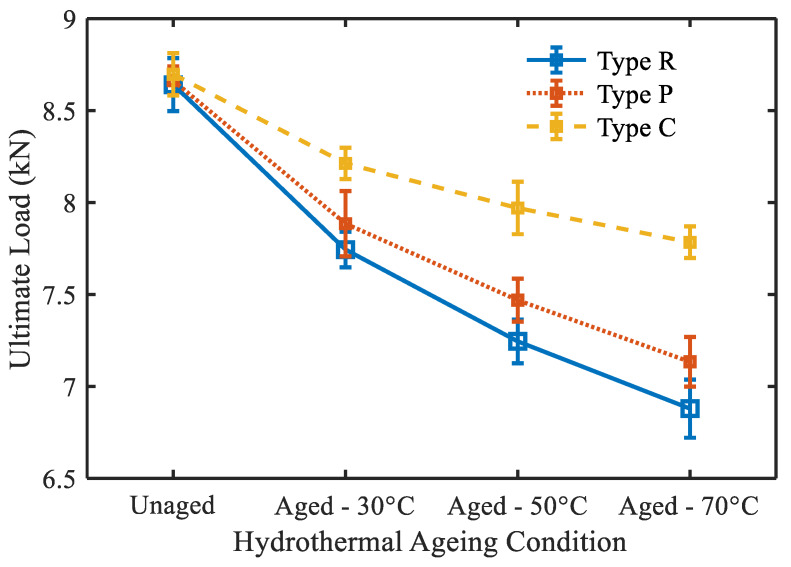
Influence of hydrothermal aging on ultimate load.

To examine the influence of hydrothermal aging on mechanical behavior, indentation tests were conducted on the dry and aged (1600 h) specimens. The indentation properties of different specimens are summed up in Table 3. As expected, it can be observed that aging caused a considerable reduction of the ultimate load and stiffness. In the aged specimens, the interfaces stuck amid fiber and matrix were the critical zone where the water molecules entered by capillarity due to the presence of some defects (Figure 10). The micro-cracks occurred at the fiber-matrix interfaces due to wetness restricted effective fiber-matrix load transfer. Compared to unmodified specimens, the mechanical property retention of specimens modified with water-resistant glass fibers was higher. From Figure 11, the Type C exhibited the greatest retention of strength and stiffness, while the Type R exhibited the least.

The immersing temperature is another vital parameter that affected the mechanical behavior of the specimens in the hydrothermal aging environment. Increasing the immersion temperature of seawater magnified its aging effect on the composites. For every temperature increment, the ultimate load and stiffness of different specimens were decreased. Specimens aged in seawater at an elevated temperature of 70 °C showed lower ultimate load and stiffness. The decrease in ultimate load and stiffness owing to the rise of immersion temperature is attributed to the weakening of the interface (Figure 10). At ambient and elevated immersion temperatures, the retention of indentation properties for different composites specimens can be graded from the lower to the higher order as: Type R < Type P < Type C.

The consequences of hydrothermal aging can be well assumed by considering the effect put forth on the individual components (fiber and matrix) of the specimens together with their interfaces. Moisture uptake led to degradation of Kevlar in the patches, stress corrosion of glass fibers [46] and weakening of the interface (see Figure 10a) [16]. Swelling of Kevlar fibers due to the continued exposure to moisture resulted in the decrement of the Kevlar fiber’s stiffness and also to the occurrence of premature debonding (shear stresses) (Figure 10a) [16,31,48,49]. The higher immersion temperature-induced higher thermal stress in the polymer matrix composites. The induced stress further enhanced the initiation and progression of micro-cracks in the interface. This event has also damaged the matrix system and hastened its aging (see Figure 10). The high water uptake is ascribed to the matrix cracks, which was noticed to be further predominant at elevated immersion temperatures. This severe weakening in resin and interface increased voids or cavities and induced the specimens to keep absorbing moisture inside. Specimens aged at immersion temperature 50 °C and especially 70 °C absorbed more moisture inside (Figure 8) and evidently, the increased moisture in the interface decreased the bond strength and resulted in uneven distribution of load.

#### 3.2.3. Effect of Moisture Absorption on AE Behavior

To examine the damage mechanisms of different specimens induced by hydrothermal aging, the AE events (transient stress wave signals) generated during the indentation tests on dry and aged specimens were examined with a conventional AE parametric analysis. In this investigation, the AE descriptor peak frequency was considered for the study of the deterioration of the composite specimens due to aging [38,50]. The AE parametric analysis was performed to show how hydrothermal aging influenced the damage distribution [51].

**Figure 12:**
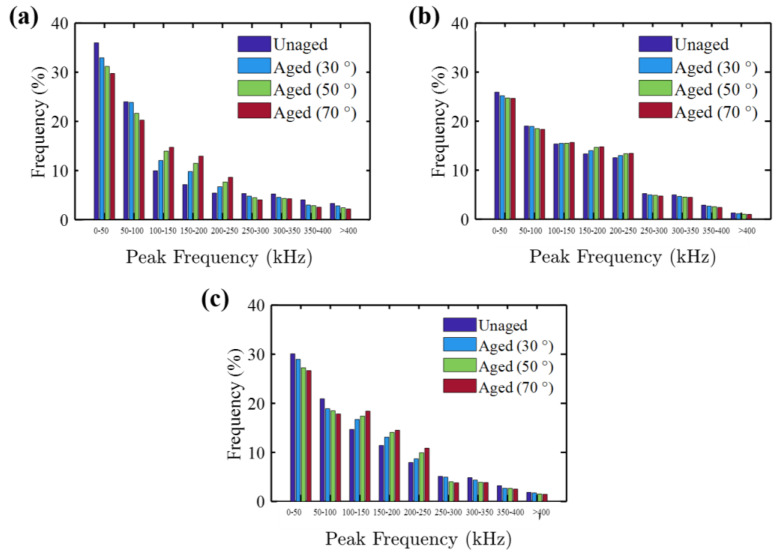
Peak frequency distribution of AE events for (**a**) Type R, (**b**) Type P and (**c**) Type C specimens.

**Figure 13:**
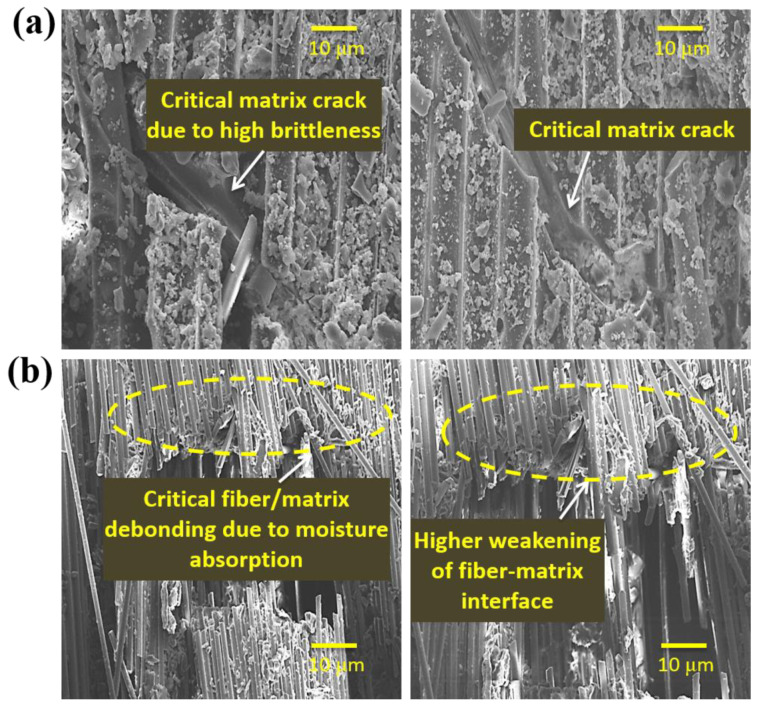
Typical SEM images showing (**a**) matrix cracking and (**b**) fiber/matrix debonding of specimens subjected to indentation load.

The percentage of AE events vs. peak frequency for the dry and aged specimens at different immersion temperature is shown in Figure 12. All the unaged specimens depicted a higher percentage of the number of AE events centered at the peak frequency around < 100 kHz. This peak frequency range is generally considered to be a feature of damage generated in the resin (e.g., micro-cracking, tearing and deformation) [52,53]. Morphological studies from SEM images support the AE test results (Figure 13a).

Hydrothermal aging caused an increase in the percentage of the number of AE events toward moderate peak frequencies (>100–250 kHz). This peak frequency range is generally considered to be the feature of the fiber/matrix interface (e.g., debonding) [48,54]. This indicates that hydrothermal aging induced more fiber-matrix interface damage such as debonding (Figure 13b). The immersion temperature, from 30 to 70 °C, induced this shift significantly. These results depict that, by the plasticization of the matrix system, the hydrothermal aging induced less damage to the matrix. The introduction of different glass fiber reinforcements seems to lessen this shift in the peak frequency distribution as regards Type R specimens. The increment in the percentage of interface debonding induced due to aging effect was significantly higher in the Type R specimens, whereas the Type C specimens exhibited the least. These results explain why Type C specimens showed a higher retention of ultimate load and stiffness.

## 4. Conclusions

In this study, the influence of hybrid patches and hydrothermal aging on indentation response of patch repairs in damaged GFRP laminates was experimentally investigated. The following conclusions were hereby drawn:

- The 50G50K specimens are the only repaired specimens that exhibited a higher indentation response (in terms of ultimate load, stiffness and permanent displacement) than the virgin ones;
- In the 50G50K, unlike virgin, 100G, and 75G25K, the Kevlar fibers restricted penetration and prevented fiber breakage. In the same way, unlike 100K and 25G75K, the glass fibers restricted delamination, bulging and elongation;
- At all immersion temperatures, moisture absorption of Type R was the highest, while the Type C exhibited the lowest. The specimens protected using particulate fibers (Type P) exhibited a response between Types R and C specimens. In all the specimens, the increase of immersion temperature reduced the duration required for attaining the saturation level in weight gain;
- At ambient and elevated immersion temperatures, the retention of indentation properties for various specimens were graded from the lower to the higher order as: Type R < Type P < Type C. For every temperature increment, the indentation performance of different specimens was reduced. Specimens aged in seawater at an elevated temperature (70 °C) recorded the lowest ultimate load and stiffness.

## Figures and Tables

**Table 1:** Configuration code and stacking sequence of different specimens considered for the hydrothermal aging study.

Specimen	Stacking Sequence	Specimen Code
Un-modified 50G50K specimens	R	Type R
50G50K specimens modified with particulate fibers	P/R/P	Type P
50G50K specimens modified with chopped fibers	C/R/C	Type C

**Table 2:** Summary of water uptake behavior of different composite specimens.

Specimen	Aging Condition (°C)	Equilibrium Moisture Content (%)	k	Diffusion Coefficient (×10^−11^ m^2^/s)
Type R	30	7.71	0.996	2.7441
	50	8.26	1.205	2.8901
	70	8.75	1.280	2.9964
Type P	30	6.18	0.860	2.5422
	50	6.68	0.9905	2.6491
	70	7.16	1.057	2.7291
Type C	30	3.15	0.439	2.2774
	50	3.49	0.477	2.3145
	70	3.68	0.489	2.3376

**Table 3:** Indentation properties of different composite specimens.

Specimen	Aging Condition	Ultimate Load (N)	Stiffness (MPa)
Type R	Unaged	8640.78	1706.50
	30 °C	7743.69	1578.45
	50 °C	7244.53	1507.16
	70 °C	6878.70	1454.88
Type P	Unaged	8662.28	1718.77
	30 °C	7884.85	1598.14
	50 °C	7468.31	1566.58
	70 °C	7133.10	1531.37
Type C	Unaged	8697.10	1739.14
	30 °C	8212.14	1644.99
	50 °C	7969.71	1621.29
	70 °C	7783.52	1594.21
